# Effect of perioperative acupoint electrical stimulation on macrophages in mice under operative stress

**DOI:** 10.1186/s12950-023-00354-x

**Published:** 2023-08-30

**Authors:** Yinzhou Zhang, Junying Wei, Xinyuan Wu, Mengting Jiang, Wuhua Ma, Yuhui Li

**Affiliations:** 1 Lingnan Medical Research Center, Guangzhou University of Chinese Medicine, Guangzhou, 510405 China; 2 The First Affiliated Hospital of Guangzhou University of Chinese Medicine, Guangzhou, 510405 China

**Keywords:** Electrical acupoint stimulation, Macrophage, Surgical stress, Perioperative period

## Abstract

The strong perioperative stress response caused by surgical anesthesia can significantly suppress immune function, and the body is in a state of immunosuppression for 3 to 4 days after surgery, which leads to an increase in the probability of postoperative infection. Traditional Chinese medicine believes that acupuncture points can "reconcile yin and yang", promote the recovery of immune function, and help reduce the incidence of postoperative infection. Macrophages are an important type of immune cells that participate in the body's innate immunity. They have powerful phagocytosis and clearance functions. They can be polarized into M1 and M2 types under the regulation of the body, and play different roles in fighting microbial infections. Among them, the M1 type can participate in the elimination of pathogens. In this study, we will investigate the perioperative acupoint electrical stimulation to alleviate the immunosuppressive state of surgical stress mice, clarify the regulation of perioperative acupoint electrical stimulation on glucocorticoids and the relationship between NF-κB molecules and macrophage polarization.The key molecules of related pathways were verified by glucocorticoid receptor inhibitors, and it was found that electrical stimulation of acupoints during the perioperative period can affect the polarization of macrophages in surgically stressed mice to the M1 type by reducing the level of glucocorticoids and promoting the expression of NF κB molecules. Further reveal the partial mechanism of electroacupuncture regulating the anti-inflammatory and pro-inflammatory processes of macrophages in the immune response.

**Supplementary Information:**

The online version contains supplementary material available at 10.1186/s12950-023-00354-x.

## Introduction

Macrophages are resident phagocytes present in lymphoid tissues.Macrophages are key effectors involved in tissue homeostasis by clearing apoptotic cells, producing growth factors, regulating inflammation and innate immunity [1]. Macrophage polarization is a process of making different functional phenotypes according to the specific surrounding environment. It is generally divided into two subgroups, namely classically activated M1 type pro-inflammatory cells and alternatively activated M2 type anti-inflammatory cells. The two extremes of transformation, but under certain circumstances, the two can be transformed into each other [2]. Preoperative anxiety, anesthesia operation, surgical trauma and other factors can trigger perioperative stress response [3]. Surgical stress can lead to the suppression of immune function. Studies have found that prostaglandins and other cytokines caused by surgical stress can cause macrophages to differentiate into M2 type, resulting in postoperative immunosuppressive state, and surgery damages macrophage-mediated immune function. Phagocytosis can adversely affect the host's innate immunity against microbial infection, which may increase the risk of secondary microbial infection after surgery [4, 5]. Our previous research found that electroacupuncture can regulate the immune maturation of dendritic cells and improve postoperative immunosuppression [6], but whether it has the same effect on macrophages is still unclear. This study intends to explore the effect of electroacupuncture on the M1/M2 polarization of macrophages under surgical stress and related mechanisms.

## Materials and methods

### Materials

#### Experimental animals

SPF -grade healthy male C57BL/6 J mice, 6–8 weeks old, were purchased from the Animal Experiment Center of Guangzhou University of Traditional Chinese Medicine, license number: SCXK (Guangdong) 2018–0034. They were kept in the Animal Center of the First Affiliated Hospital of Guangzhou University of Traditional Chinese Medicine and given free access to food and water. Experiments were started after one week of adaptive feeding. All animal experiments in this experiment complied with the requirements of the Animal Experiment Ethics Committee. It has passed the ethical review of experimental animals in the First Affiliated Hospital of Guangzhou University of Traditional Chinese Medicine, number: GZTCMF1-2,022,122.

#### Main experimental reagents and instruments

FITC Anti Mouse F4/80 and APC Anti Mouse CD86 were purchased from e Bioscience Company in the United States, PE Anti Mouse CD206 was purchased from Biolagend Company in the United States, PBS was purchased from Thermo Fisher Company in the United States; red blood cell lysate was purchased from Beijing Soleil Bao Biotechnology Company; 200 -mesh cell screen and 50 ml centrifuge tube were purchased from Zhejiang Shuohua Biotechnology Company; flow cytometry tubes were purchased from BD Company in the United States; CORT E lisa kit was purchased from Cayman Company in the United Kingdom; TRI zol reagent was purchased from From Invitrogen, USA; E vo M -MLV reverse transcription kit II, SYBR Green Pro Taq HS premixed type qPCR kits and ROX dyes were purchased from Hunan Aikerui Company; primers were provided by Shanghai Bioengineering Company; RIPA lysate ( enhanced), BCA protein quantification kit ( enhanced), Loading Buffer All provided by Shanghai Biyuntian Company; rabbit source Anti Mouse NF -κB P65 primary antibody, rabbit source Anti Mouse NF-κB p P65 primary antibody, rabbit source Anti Mouse IKB-α primary antibody, rabbit source Anti Mouse p IKB-α primary antibody was purchased from Abcam company in the UK, rabbit source Anti Mouse The α—tubulin primary antibody and the rabbit-derived Anti Mouse GR primary antibody were purchased from Changzhou Affinity, the goat anti-rabbit secondary antibody was purchased from Wuhan Proteintech Company, and the ECL hypersensitive chemiluminescent reagent was purchased from Millipore Company in the United States. The brand of flow cytometer is Novo press, the brand of microplate reader is Bio-Tek, the brand of electrophoresis and developing equipment is Bio Rad, and the brand of amplification instrument is ViiA 7.

### Experimental method

#### Establishment of surgical stress model

Refer to the literature to establish the surgical stress model [7]. This model is widely used, and its advantage is that it causes less trauma to the mice, has little impact on the vital signs of the mice, and has a slight impact on other organs, making the model stable and easy to replicate. The specific operation was as follows: After the mice were fasted for 12 h, they were anesthetized and fixed with 2% sevoflurane, and the abdominal cavity was explored gently to prevent intestinal bleeding or damage to abdominal blood vessels. The operation lasted about 10 min. After the exploration, the muscle fascia and skin were sutured, and aseptic operation was guaranteed throughout the operation. The 42 mice were divided into 7 groups according to the random number table method, 6 in each group, respectively blank control (C) group, 2 h operation group, 6 h operation group, 12 h operation group, 24 h operation group, 48 h operation group, and 72 h operation group. surgery group. The experimental animals were sacrificed by cervical dislocation at 2 h, 6 h, 12 h, 24 h, 48 h, and 72 h after operation. The spleen was obtained, and the percentages of CD86 and CD206 on the surface of macrophages were detected by flow cytometry to select the time point for sampling after operation.

#### Preparation of spleen single cell suspension

After the animal was sacrificed, the mouse chest cavity was opened to expose the heart, the right atrial appendage was cut open, a 20 ml syringe was inserted into the left ventricle, and normal saline was injected slowly for perfusion until the liver turned white. Open the abdominal cavity, take out the spleen and place it in a petri dish with pre-cooled PBS, and peel off the connective tissue. Afterwards, the spleen was ground on a 200- mesh cell sieve, and at the same time, it was washed with PBS to collect the spleen tissue cell suspension. The resulting cell suspension was filtered through a 200- mesh cell sieve again and then centrifuged (1700 rpm, 5 min. The same below). Discard the supernatant, add 5 ml erythrocyte lysate and let it stand on ice for 10 min, during which time, turn it upside down twice to fully lyse it, and centrifuge again to discard the supernatant. Add 2 ml PBS to resuspend the cells.

#### Detecting the expression of CD86 and CD206 on the surface of macrophages by flow cytometry

Draw 100 ul of cell suspension into the flow cytometry tube, add flow cytometry antibodies FITC Anti Mouse F4/80, APC Anti Mouse CD86, PE Anti Mouse CD206, shake and mix well, and incubate on ice in the dark for 30 min. Add 2 ml PBS to wash twice, centrifuge to discard the supernatant, add 500 ul PBS to resuspend, and use flow cytometry for detection.

### Explore the regulatory effect of electroacupuncture on surgical stress and macrophages

#### Experimental grouping and electroacupuncture adjustment methods

Part I: 24 mice were randomly divided into 4 groups, 6 in each group, namely blank control (C) group, operation ( S) group, electroacupuncture (E) group, and non-acupoint electroacupuncture (N) group. According to the " Acupoint Location Standards for Experimental Animals ", the mouse standard acupoints were located. After the acupoints were selected, 2/15 Hz sparse-dense wave electroacupuncture was given with a current of 1 mA. The electroacupuncture group and the non-acupoint electroacupuncture group were given one electroacupuncture stimulation 12 h before operation after anesthesia, and the other groups were only anesthetized without operation, and the electroacupuncture stimulation time was 30 min. Re- anesthetized and given electroacupuncture stimulation 10 min before the operation, the state of electroacupuncture stimulation was maintained during the operation, and the entire electroacupuncture stimulation time was 30 min. The stimulation sites in the electroacupuncture group were Zusanli and Sanyinjiao, and the stimulation sites in the non-acupoint electroacupuncture group were the buttock muscles 3 mm away from the depression between the base of the tail and the anus. Group C only underwent fixation, skin preparation, and povidone-iodine disinfection; Group S only underwent exploratory laparotomy without electroacupuncture stimulation.

Part II: 30 mice were randomly divided into 5 groups, 6 in each group, respectively blank control group (CON group), blank control + DMSO group (CD group), operation + DMSO group (SD group), operation + RU486 group (SR group), electroacupuncture group (EA group). The mice were weighed and recorded, and administered at 20 mg/kg. A certain amount of DMSO solution was injected intraperitoneally after anesthesia in the CD and SD groups 12 h before the operation, and the same amount of DMSO solution before the operation was injected again in the SD group 20 min after the operation, and then put back into the cage. In the SR group, the mice were anesthetized 12 h before the operation, and a certain amount of RU486 solution was injected intraperitoneally according to the weight of the mice, and then the mice were placed on the heat preservation pad and returned to the cage after they woke up naturally, and fasted. The SD and SR groups underwent exploratory laparotomy, and the EA group underwent electroacupuncture stimulation. The method was the same as the first part.

**Fig. 1:**
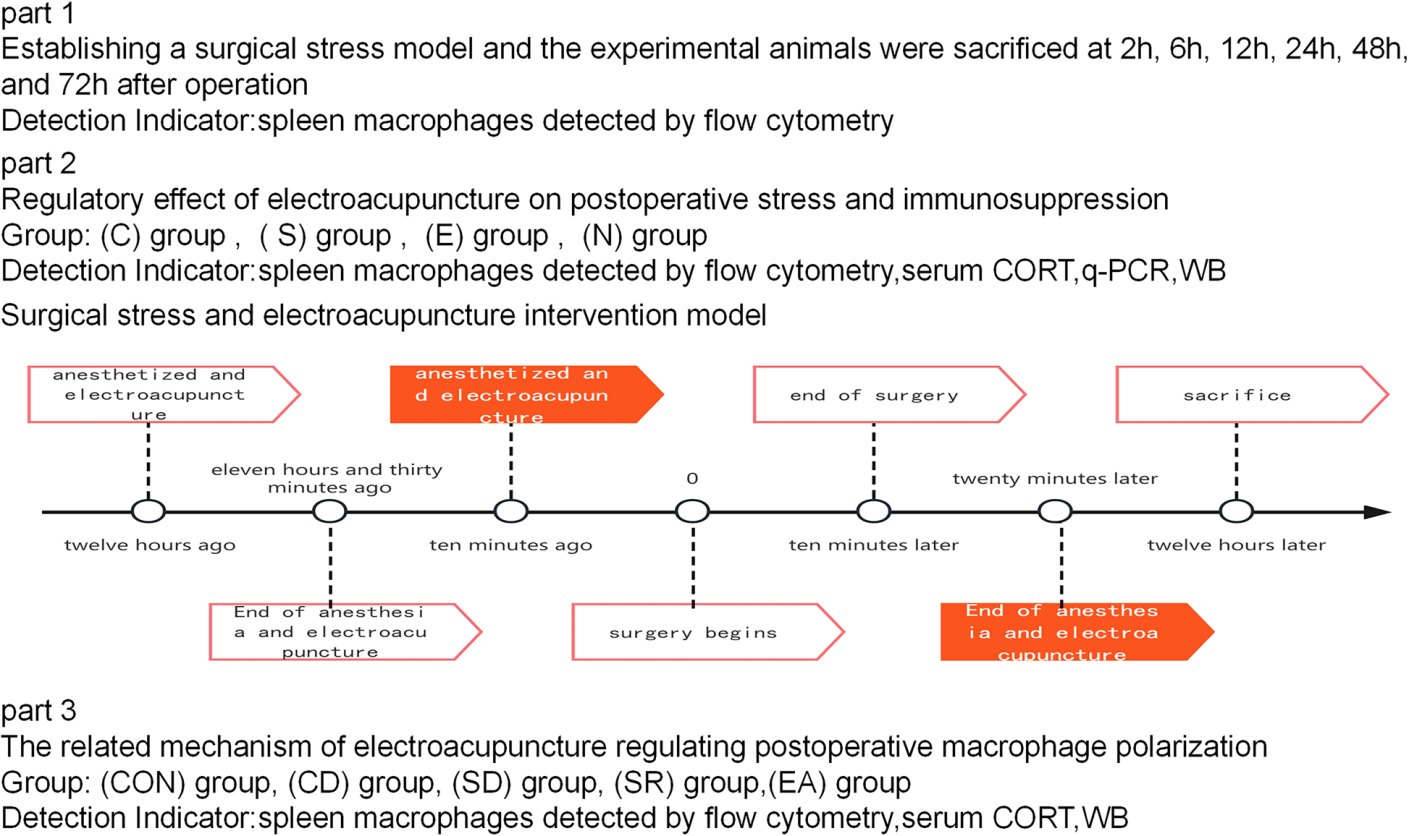
Work flow

According to the above experimental results, the sample was collected at 12 h. The eyeballs were removed first to take blood. Afterwards, the mice were sacrificed by cervical dislocation, soaked in alcohol for 1 min, and then the chest cavity was opened for cardiac perfusion. After the liver turned white, the abdominal cavity was opened to take the spleen. Figure 1 is a work flow chart.

#### Detection of the percentage of CD86 + , CD206 + cells on the surface of macrophages by flow cytometry

The spleen was placed in a pre-cooled PBS culture dish, the connective tissue was peeled off, and part of the spleen was placed on a 200 -mesh cell sieve and thoroughly ground to obtain a spleen cell suspension. The remaining spleen tissue was stored in a -80 °C refrigerator. Take the resuspended splenocyte suspension and add flow cytometry antibodies FITC Anti Mouse F4/80, APC Anti Mouse CD86, PE Anti M ouse CD206, incubate on ice for 30 min, wash with 2 ml PBS, and centrifuge. After the cells were resuspended in 500 ul PBS, the percentages of CD86 + and CD206 + cells on the surface of macrophages were detected by flow cytometry.

#### Detecting the content of corticosterone (CORT) in mouse serum by Elisa

After removing the eyeball and taking blood, place the blood in a refrigerator at 4 °C overnight, centrifuge (3000 rpm, 15 min) and aspirate the supernatant. The CORT E lisa kit was used for detection, and the operation was carried out according to the instructions. The microplate reader detected the OD value of CORT in the serum of each group of mice, and the original software was used to calculate the content of CORT in peripheral blood.

#### q -PCR detection of carbon monoxide synthase (INOS), arginase -1 (A rg -1), TNF-α, IL—10, IL -1βm RNA expression in spleen

Take part of the spleen tissue and put it into a homogenization tube after weighing, add magnetic beads, add a certain amount of TRIzol according to the weight of the spleen, and place it in a homogenizer to fully homogenize and lyse to extract RNA. The concentration of extracted RNA was detected by UV spectrophotometer. Reverse transcription was performed using Evo M-MLV kit II, and the cDNA of the reverse transcription product was stored in a -20 °C refrigerator for later use. Take 1 ug cDNA according to SYBR Green Pro T aq HS premix _ The qPCR kit instructions were used to calculate the 2^ (-∆∆)CT value. The primers used in the experiment were: β-actin (F: 5'-GTCGTACCACAGGCATTGTGATGG-3', R: 5'-GCAATGCCTGGGTACATGGTGG-3'), INOS (F: 5'- ATTTTGCATGACACTCTTCACCCAC -3', R: 5'- TAGGCTTGTCTCTGGGTCCTCT—3'), Arg -1 (F: 5'- CATATCTGCCAAAGACATCGTG -3', R: 5'- GACATCAAAGCTCAGGTGAATC -3') TNF-α (F: 5'-ATGTCTCAGCCCTCTTCTCATTC-3', R: 5'-GCTTGTCACTCGAATTTTGAGA- 3') IL-10 (F: 5'- TTCTTTCAAACAAAGGACCAGC -3',R: 5'- GCAACCCAAGTAACCCTTAAAG -3'), IL-1β (F: 5'-GCTTCCAAACCTTTGACCTG-3',R: 5'-CTGTTGTTTCCCAGGAAGAC-3 ').

#### Detection of expression of spleen GR, NF-κB P65, NF-κB p -P65, IKBα, p -IKBα protein by Western Blot

Take part of the spleen tissue and put it into a homogenization tube after weighing, add magnetic beads, add RIPA lysate according to the weight of the spleen, and place it in a homogenizer to fully homogenize and lyse. After lysis, let stand on ice for 30 min and then centrifuge (15,000 rpm, 30 min), draw the supernatant into a clean EP tube, and use BCA kit for protein quantification. After adjusting the protein concentration to be consistent, add protein loading buffer and heat in a water bath at 100 °C for 10 min to denature the protein, then perform SDS-PAGE gel electrophoresis to separate the target protein and transfer it to a PVDF membrane, 5 Block with % skimmed milk at room temperature for 1 h, wash with TBST three times, add primary antibodies to GR, NF-κB p65, NF-κB p -P65, IKBα, p -IKBα, and α- tubulin, and incubate overnight in a refrigerator at 4 °C. Recover primary antibody, wash 3 times with TBST, add HRP- labeled goat anti-rabbit IgG secondary antibody, incubate at room temperature for 1 h, recover secondary antibody, wash 3 times with TBST, develop with ECL ultra-sensitive chemiluminescent solution, and use Image Lab software Analyze band gray values.

#### Statistical processing

SPSS 25 statistical software was used for statistical analysis. The measurement data of normal distribution were expressed as mean ± standard deviation ( ‾x ± s). When the variances were equal, the comparison of the means of multiple samples was performed by one-way analysis of variance. LSD- t method was used for comparison. Welchs's ANOVA was used to compare the means of multiple samples when the variances were not homogeneous, and the Games-Howell test method was used for pairwise comparisons between groups. *P* < 0.05 considered the difference to be statistically significant.

## Results

**Fig. 2:**
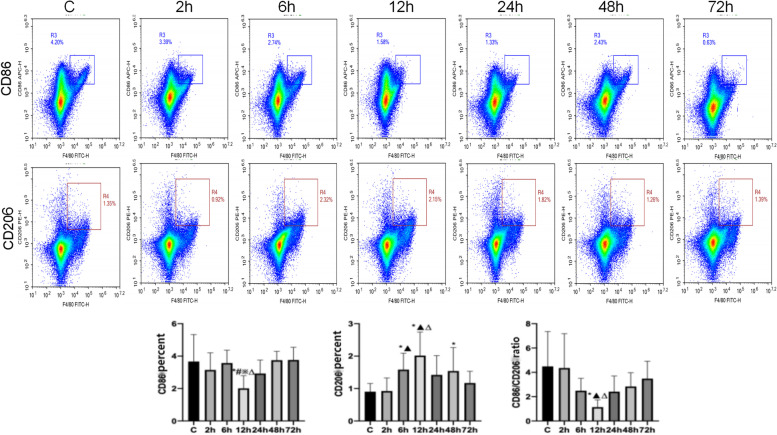
Percentage of CD86 + and CD206 + cells at each time point after operation, CD86 + /CD206 + ratio. Note: Compared with group C, * *P* < 0.05; compared with 2 h, ∆ *P* < 0.05; compared with 6 h, # *P* < 0.05; compared with 48 h, ※ *P* < 0.05; compared with 72 h, ▲ *P* < 0.05

The expression of CD86 and CD206 at each time point after operation with group C, the percentages of CD86 + cells in the 2 h, 6 h, 12 h,24 h, 48 h, and 72 h operation groups were lower than those in group C, but the difference was not statistically significant (*P* > 0.05); the CD86 + cells in the 12 h operation group The percentage of cells decreased compared with group C, 6 h, 48 h, and 72 h operation groups ( *P* < 0.05); compared with group C, the percentages of CD206 + cells in 6 h, 12 h, and 48 h operation groups all increased ( *P* < 0.05), and the percentage of CD 206 + cells in the 12 h operation group was higher than that in the 2 h and 72 h groups ( *P* < 0.05); compared with group C, 2 h and 72 h operation groups, the The ratios of CD86 + /CD206 + cells were all decreased ( *P* < 0.05) (Fig. 2).

### Regulatory effect of electroacupuncture on postoperative stress and immunosuppression

**Fig. 3:**
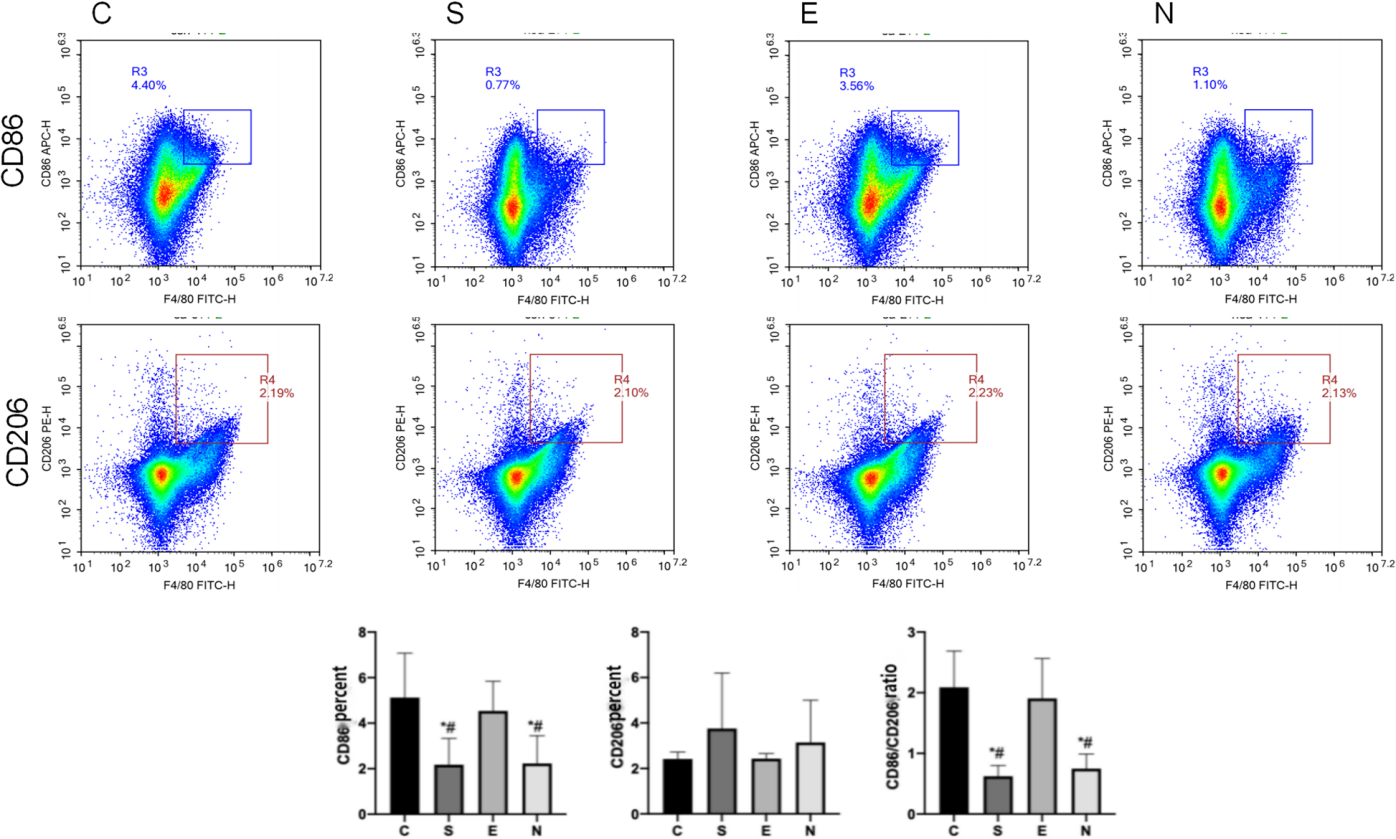
Each group CD86 + , CD206 + cell percentage, CD86 + /CD206 + ratio. Note: *: Compared with group C, *P* < 0.05; #: Compared with group E, *P* < 0.05

Expression of CD86 and CD206 on the surface of splenic macrophages with group C, the percentage of CD86 + cells on the surface of macrophages and the ratio of CD86 + /CD206 + in group S and group N were all decreased ( *P* < 0.05), and there was no significant difference in each index in group E (*P* > 0.05); compared with group S, the percentage of CD86 + cells on the surface of macrophages and the ratio of CD86 + /CD206 + in group E increased ( *P* < 0.05), and there was no significant difference in each index in group N (*P* > 0.05); compared with group E, the surface of macrophages in group N The percentage of CD86 + cells and the ratio of CD86 + /CD206 + decreased ( *P* < 0.05) (Fig. 3).

**Fig. 4:**
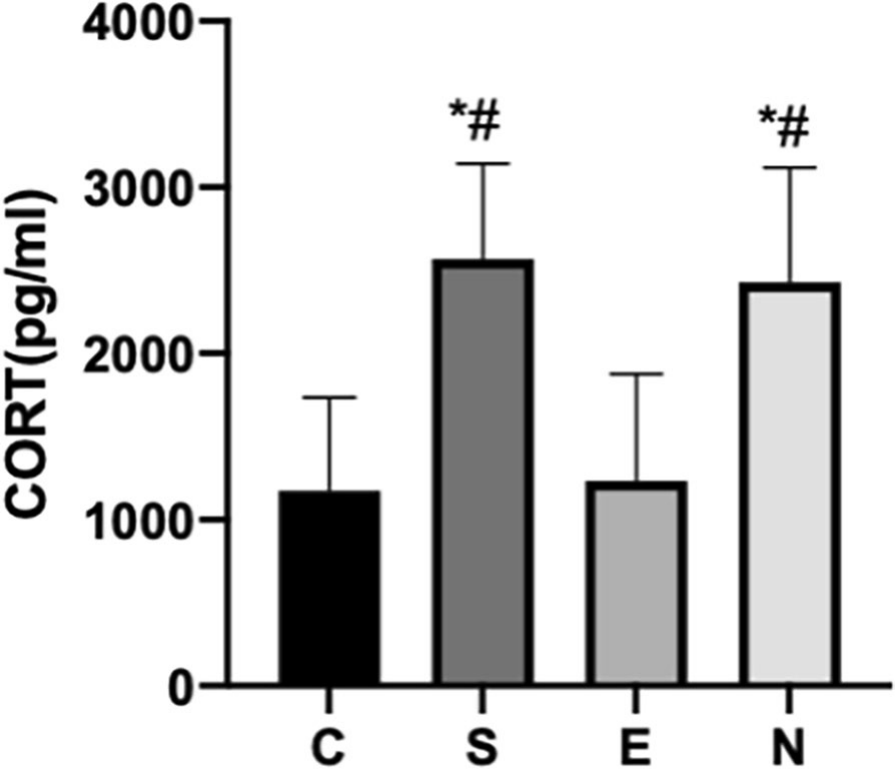
The content of CORT in the serum of each group. Note: *: Compared with group C, *P* < 0.05; #: Compared with group E, *P* < 0.05

Serum CORT content with group C, the levels of CORT in peripheral blood of group S and group N were significantly increased (*P* < 0.05), and the level of CORT in peripheral blood of group E was not significantly increased (*P* > 0.05); The content of CORT in the blood was significantly lower, and the CORT content in the peripheral blood of the N group was not significantly different from that of the S group (*P* > 0.05). Compared with the E group, the CORT content of the peripheral blood of the N group was significantly increased (*P* < 0.05) (Fig. 4).

**Fig. 5:**
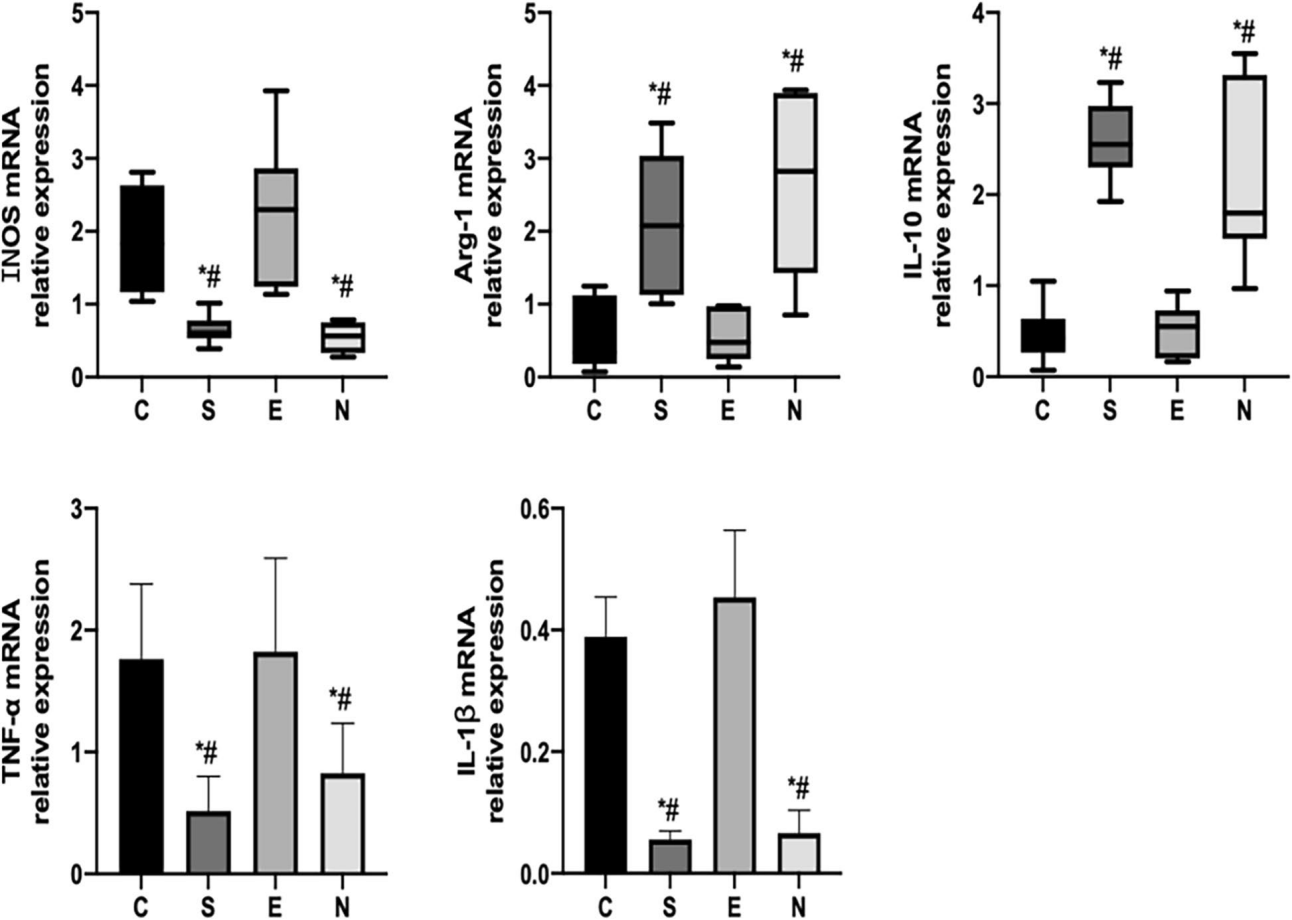
Relative expression of mRNA of INOS, Arg—1, TNF-α, IL -1β, IL—10 in each group. Note: *: Compared with group C, *P* < 0.05; #: Compared with group E, *P* < 0.05

Expression of INOS, Arg -1, TNF-α, IL -1β, IL—10 mRNA with group C, the expressions of Arg -1 and IL—10 mRNA in groups S and N increased ( *P* < 0.05), while the expressions of INOS, TNF-α and IL—1β mRNA decreased (*P* < 0.05), each index of Group E There was no significant change in mRNA expression (*P* > 0.05); compared with group S, the expression of Arg -1 and IL—10 mRNA in group E decreased (*P* < 0.05), and the expression of INOS, TNF-α, IL -1βm RNA The expressions of all increased (*P* < 0.05), each index in group N There was no significant change in mRNA expression (*P* > 0.05); compared with group E, the expressions of Arg -1 and IL-10 mRNA in group N were all increased ( *P* < 0.05), INOS, TNF-α, IL -1β The expression of m RN decreased (*P* < 0.05) (Fig. 5).

**Fig. 6:**
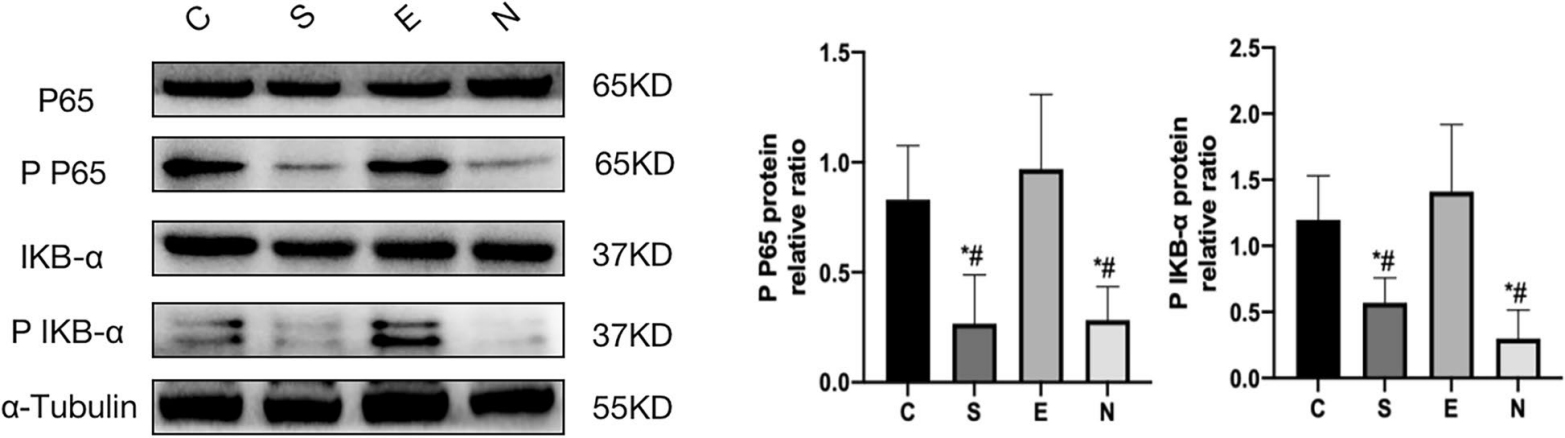
NF-κB P65, NF-κB p P65, IKB-α, P IKB-α protein expression in each group. Note: *: Compared with group C, *P* < 0.05; #: Compared with group E, *P* < 0.05

Expression of NF-κB p65, NF-κB p -P65, IKBα, p -IKBα protein with group C, the expressions of NF-κB p -P65 and p -IKBα proteins in groups S and N were all decreased (*P* < 0.05), and the expressions of NF-κB p -P65 and p -IKBα proteins in group E had no significant difference (*P* > 0.05); compared with group S, the expressions of NF-κB p -P65 and p -IKBα proteins in group E increased (*P* < 0.05), while the expressions of NF-κB p -P65 and p -IKBα proteins in group N had no significant difference (*P* > 0.05); Compared with group E, the expression of NF-κB p -P65 and p -IKBα proteins in group N increased (*P* < 0.05) (Fig. 6).

### The related mechanism of electroacupuncture regulating postoperative macrophage polarization

#### Expression of CD86 and CD206 on the surface of splenic macrophages (Fig. 7)

**Fig. 7:**
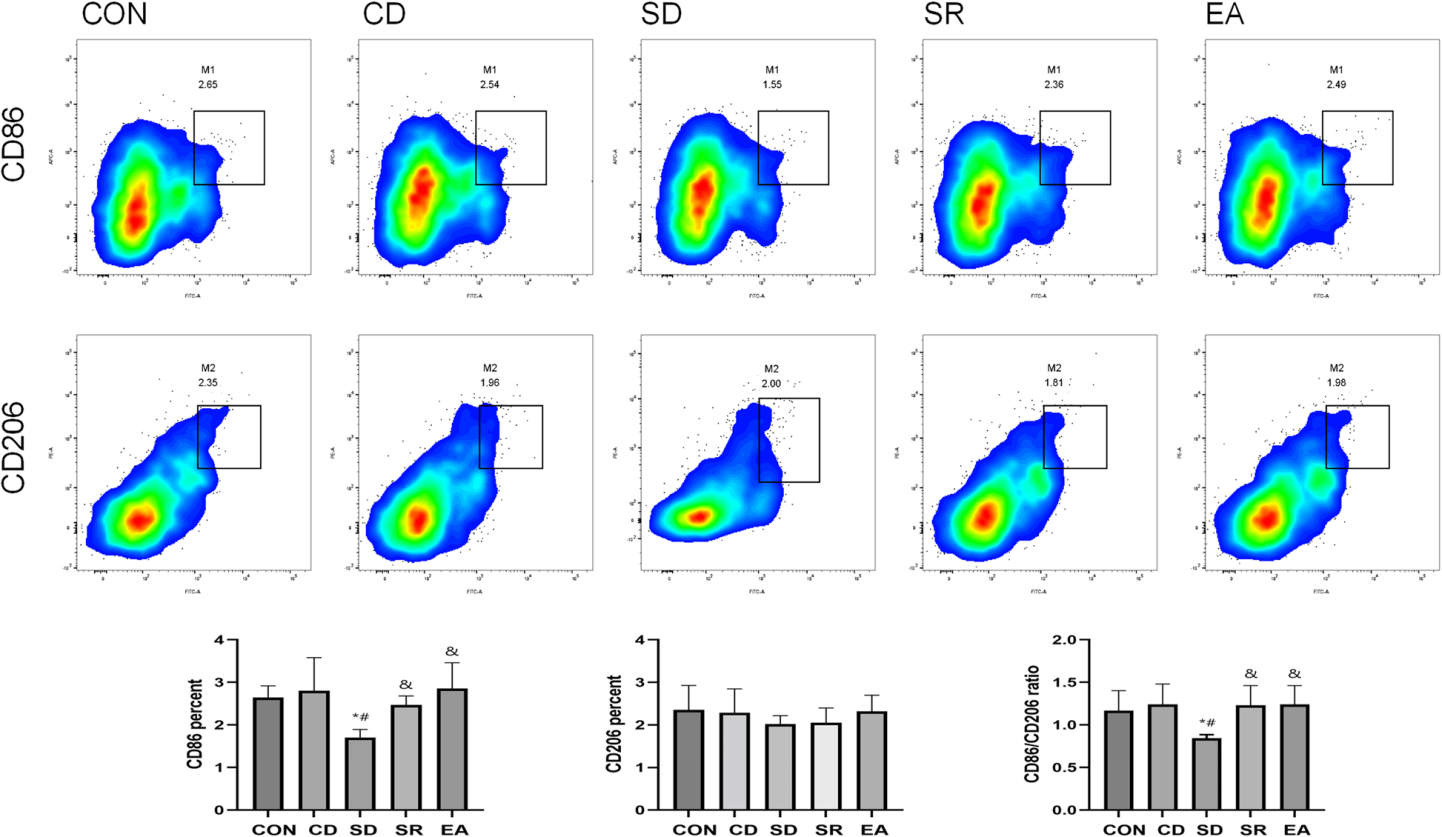
Each group CD86 + , CD206 + cell percentage, CD86 + /CD206 + ratio. Note: *: Compared with CON group, *P* < 0.05; #: Compared with CD group, *P* < 0.05; &: Compared with SD group, *P* < 0.05

#### Serum CORT content (Fig. 8)

**Fig. 8:**
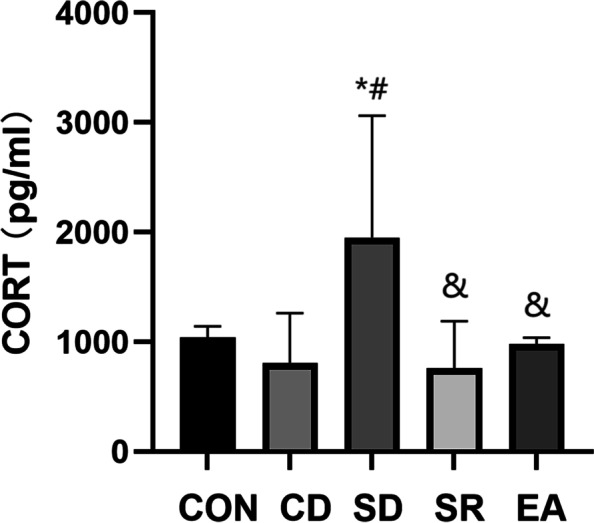
The content of CORT in the serum of each group. Note: *: Compared with CON group, *P* < 0.05; #: Compared with CD group, *P* < 0.05; &: Compared with SD group, *P* < 0.05

#### Expression of GR, NF-κB P65, NF-κB p -P65 protein (Fig. 9)

**Fig. 9:**
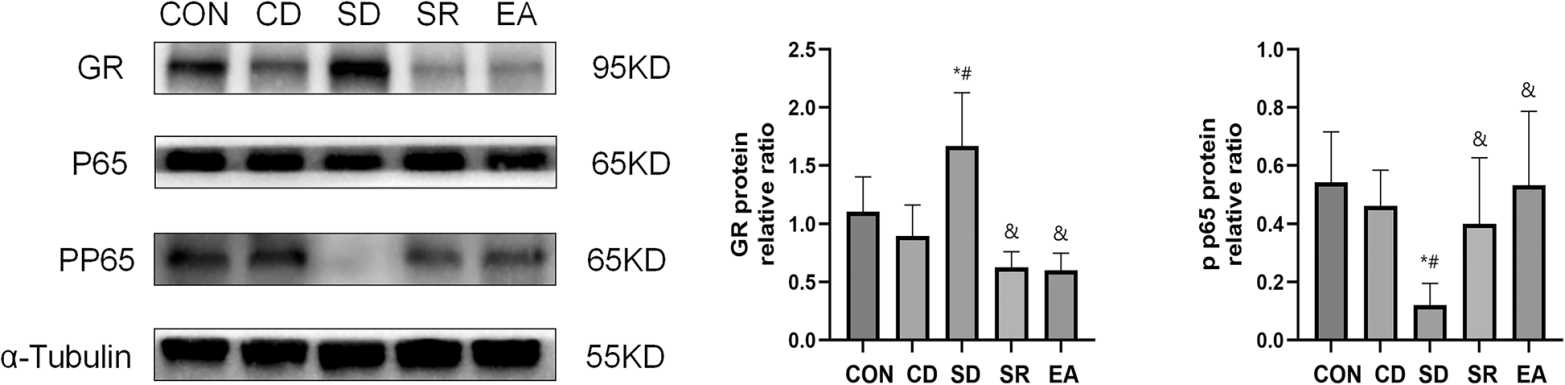
GR, NF-κB P65, NF-κB p P65 protein expression in each group. *: Compared with CON group, *P* < 0.05; #: Compared with CD group, *P* < 0.05; &: Compared with SD group, *P* < 0.05

## Discussion

Extensive cell-mediated immunodeficiency was observed after surgery, including chemotaxis of neutrophils, macrophage-associated immune responses, natural killer cell activity, lymphocyte proliferation, and decreased function, which is a symptom of surgery-induced postoperative immunodeficiency. Features of suppression [8]. The immune changes that occur during the perioperative period are mainly surgical trauma and subsequent neuroendocrine responses [2]. Activation of the hypothalamic—pituitary—adrenal axis (HPA), a key response to stress, plays a central role in mediating the effects of surgery on the immune system [9], with increased production of ACTH from the pituitary gland and subsequent release of ACTH from the adrenal cortex. Endogenous glucocorticoids (GC), cortisol in mammals and CORT in rodents. The effect of GC on macrophages can directly inhibit their immune activity [10]. Some studies have shown that GC weakens the gene expression changes induced by almost all traumas, thereby inhibiting the differentiation of macrophages into M1 phenotype and producing anti-macrophages. Immunosuppression of phagocytes [11]. In addition, GCs can also polarize macrophages into an M2-like phenotype by regulating the expression of anti-inflammatory proteins [12].

Macrophages are the sentinel cells of immune function and the key cells connecting innate and adaptive immune responses. Inflammation triggers the recruitment and differentiation of monocytes in the blood circulation into macrophages [1], which engulf pathogenic microorganisms and transmit antigen signals Initiates an adaptive immune response to T cells. Resident macrophages reside in major immune organs such as the spleen, and play important roles in tissue stabilization, such as eliminating infectious agents, mediating inflammatory responses, and repairing tissue damage [13]. Macrophage polarization is determined by both genetic and environmental factors. The polarization of macrophages determines the type of immune response, but under certain circumstances, the two can be transformed into each other [2]. M 1 macrophages express high levels of inducible INOS, TNF-α, IL-1β, IL- 6, etc. to induce the body's pro-inflammatory response. On the other hand, IL -10, IL -13 and GC can all induce monocyte cells become M2 macrophages, and M2 macrophages secrete high levels of IL -10, Arg -1, etc. at the same time to play an anti—inflammatory role [14]. Studies have found that stress hormones released under stress conditions, such as CORT, can inhibit the polarization of macrophages to M1, and down-regulate inflammatory cytokines and chemokines that polarize M1 macrophages. It is more likely to cause the polarization of macrophages to M2, resulting in impaired inflammatory response and promoting the spread of the disease [15].

NF-κB is a key transcription factor of stress and injury in eukaryotic cells, which is involved in normal activities and disease processes of the body, involving growth and development, cell differentiation, apoptosis, immune response, inflammatory response, trauma, stress Inflammation, tumor growth, chronic inflammatory diseases and other processes [16, 17]. NF-κB can be activated by a variety of stimuli, it is the central regulator of innate immunity and acquired immunity, it can regulate the expression of various pro-inflammatory factors and anti-apoptotic proteins[16], and its nuclear translocation appears in normal animals Daily rhythm, the transcriptional activity of NF-κB is inhibited by increasing endogenous corticosterone under mild stress [17]. Current research shows that glucocorticoid induces NF-κB gene transcription in two ways, one is through protein–protein interaction, in the nucleus, glucocorticoid-glucocorticoid receptor complex and NF- κB active p65 subunit of κB interacts and prevents the binding of transcriptionally active NF-κB and DNA recognition structures in the promoter region of the gene of interest. The second mode of action is that glucocorticoids induce the expression of IκB. Active NF-κB is subsequently captured, which in turn turns off NF-κB- driven gene transcription. The obvious decrease in the expression of NF-κB observed in the surgical trauma model rats [18] may be related to the increase of endogenous glucocorticoids. Activation of NF-κB in macrophages will promote the differentiation of M1 macrophages, upregulate related downstream genes, and increase the expression of M1 representative pro-inflammatory cytokines IL-6 and TNF-α [19]. Therefore, during the perioperative period, it is of great significance to suppress the body's stress response, reduce the expression of glucocorticoids, and activate the transcription of NF-κB to polarize macrophages to the M1 type, thereby enhancing the body's immune function and reducing the risk of postoperative infection.

The immunomodulatory effect of electroacupuncture has been widely recognized. Electroacupuncture can prevent postoperative hyperglycemia by inhibiting ACTH, and improve the stress response and immune function of patients undergoing general anesthesia [20]. Zusanli is the lower He acupoint of the Stomach Meridian of Foot Yangming. It is a meridian of plethora of qi and blood. It can regulate the flow of qi and reduce adverse qi. At the same time, Zusanli stimulates and activates the afferent sciatic nerve, which innervates the side of the mouth through the nerve next to the trigeminal nerve. Therefore, the ventrolateral nucleus can inhibit the secretion of CRH through the paraventricular nucleus of the hypothalamus, inhibit the generation of ACTH in the pituitary gland [21], and thus inhibit the secretion of CORT. Sanyinjiao is one of the important acupoints of the Spleen Meridian of Foot Taiyin. It is the meridian of excess blood and little Qi. It cooperates with Zusanli to regulate yin and yang in the body and enhance the body's resistance [22]. In terms of the selection of electroacupuncture frequency, Han Jisheng [23] et al. found that the alternating density wave stimulation of 2/15 Hz can simultaneously stimulate the release of dynorphin and endomorphin to achieve a good analgesic effect and reduce the stress response. In addition, there are evidences from different centers that electroacupuncture can regulate inflammatory response by regulating NF-κB pathway [24, 25]. Electroacupuncture stimulation of Zusanli point in rats with surgical trauma can significantly up-regulate the expression of NF-κB, and improve postoperative immunosuppression by up-regulating NF-κB activity [26]. This study is a continuation of the previous study [6] to select the combination of Zusanli and Sanyinjiao acupoints to inhibit the body's stress response, activate the NF- κB pathway, and improve postoperative immunosuppression.

In this study, by comparing the percentage of CD86 + cells on the surface of macrophages, the percentage of CD206 + cells and the ratio of CD86 + /CD206 + cells at 2 h, 6 h, 12 h, 24 h, 48 h, and 72 h after surgery, it was found that postoperative The percentage of CD86 + cells and the ratio of CD86 + /CD206 + decreased at 2 h, and the decrease was the most obvious at 12 h after operation, and did not return to the normal level at 72 h after operation, indicating that the surgical stress response under general anesthesia can significantly inhibit the giant cells. Macrophages are polarized towards the M1 type. Because the resident macrophages exist in the main immune organs such as the spleen, this study chose to take the spleen 12 h after the operation. Elisa results showed that postoperative serum CORT levels in group E were significantly lower than those in group S, indicating that electrical acupoint stimulation during the perioperative period can reduce the stress response of surgery, which is consistent with other studies [24]. The level of CORT in group E was significantly lower than that in group N, but there was no significant difference in the level of CORT between group N and group S, indicating that the stimulating effect of electroacupuncture is related to acupoints. The results of qPCR showed that Arg—1, IL -10 in the spleen The expression of mRNA was lower in group E, and the expression of INOS, TNF-α, and IL-1β cytokines in group E was higher than that in group S and group N, indicating that electroacupuncture stimulation can increase the expression of cytokines in M1 macrophages after operation. Secretion, promotes the polarization of macrophages to the M1 type, and promotes postoperative immune response, and the stimulating effect of electroacupuncture is related to acupoints. In order to further clarify whether perioperative acupoint electrical stimulation regulates macrophage polarization through GR and its downstream molecule NF-κB pathway, we set up a surgery + RU486 group for verification, and DMSO solvent without RU486 group as a control. The results of western blot showed that the level of NF-κB p -P65 in the SD group was significantly reduced after operation, and the expression of NF-κB p -P65 in the SR group and the electroacupuncture group could significantly increase after operation, indicating that electroacupuncture can reduce the level of glucocorticoids and promote NF-κB transcription, indicating that electroacupuncture regulates the polarization of M1 macrophages is related to the NF-κB pathway, and this regulation of electroacupuncture has acupoint specificity.

In summary, electroacupuncture can inhibit the stress response of surgery, reduce the expression of CORT after surgery, and reduce the inhibitory effect of surgical stress on the polarization of M1 macrophages through the NF-κB molecular pathway, and promote the recovery of postoperative immune function, and the stimulating effect of electroacupuncture is specific to acupoints.

However, our research still has some limitations. Although the surgical stress model we used can improve the immunosuppressive effect of macrophages after electroacupuncture intervention, we did not further explore whether macrophages have postoperative anti-infective ability. It has been improved, and there is a lack of sufficient cell experiments to verify the pathway mechanism. Therefore, our future research will focus on whether electrical stimulation of acupoints during the perioperative period can enhance the anti-infection ability of macrophages and verify the key pathways at the cellular level.

### Supplementary Information

**Additional file 1. **

## Data Availability

Not applicable.
