# Supplementary material for: Effect of perioperative acupoint electrical stimulation on macrophages in mice under operative stress
Source: J Inflamm (Lond). 2023 Aug 30;20:29. doi: 10.1186/s12950-023-00354-x (PMC10470139; doi:10.1186/s12950-023-00354-x)

C S E N

P65
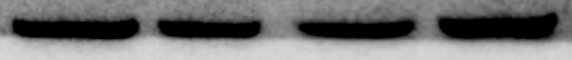


α-tubulin
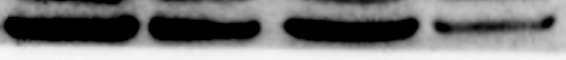


C S E N

P65
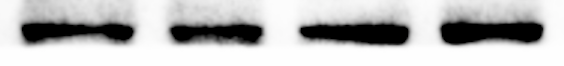


α-tubulin
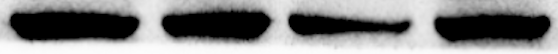


C S E N C S E N

P65
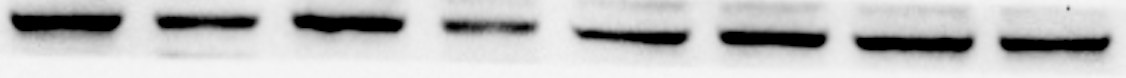


1. tubulin
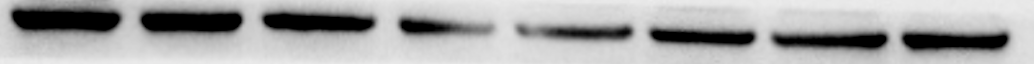


C S E N C S E N

P65


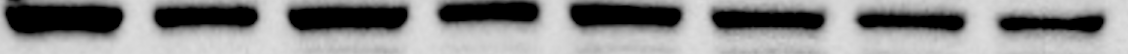


a-tubulin


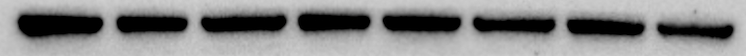


C S E N C S E N

P-P65


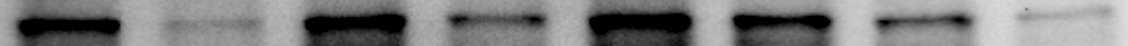


a-tubulin


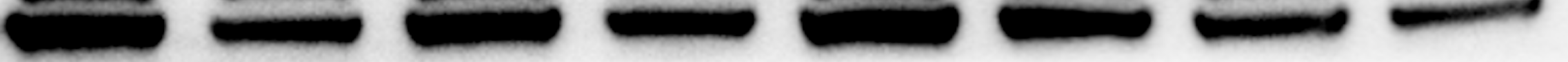


C S E N C S E N

P-P65


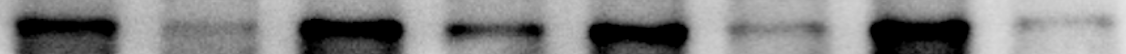


a-tubulin


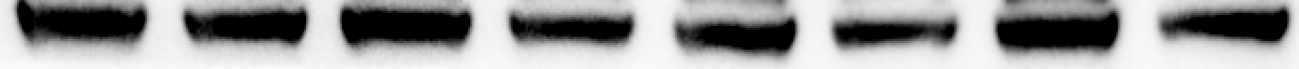


C S E N

P-P65
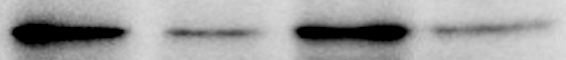


α-tubulin
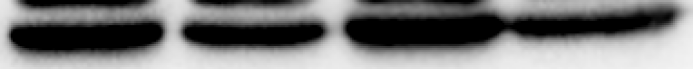


C S E N

P-P65
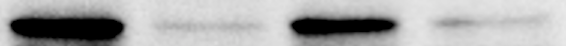


α-tubulin
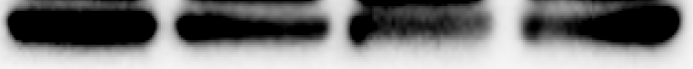


C S E N

IKB
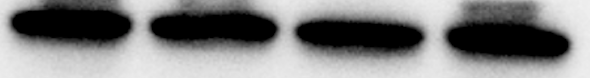


α-tubulin
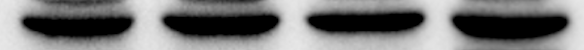


C S E N

IKB
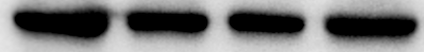


α-tubulin
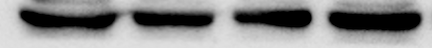


C S E N

IKB
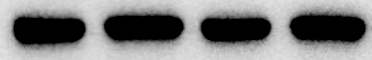


α-tubulin
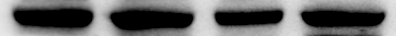


C S E N C S E N

IKB
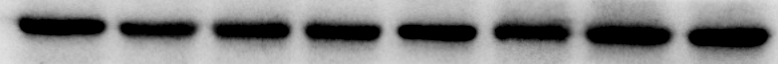


a-tubulin


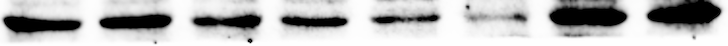


C S E N

IKB
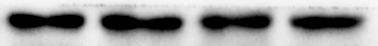


α-tubulin
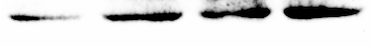


C S E N

p-IKB
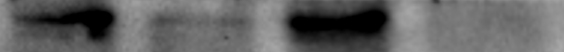


α-tubulin
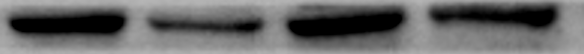


C S E N

P-IKB
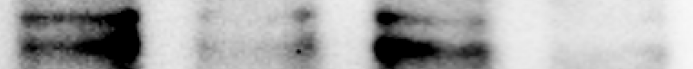


α-tubulin
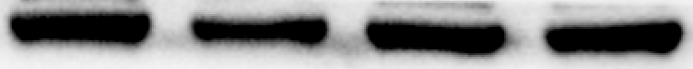


C S E N

P-IKB
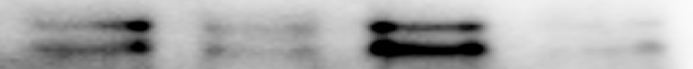


α-tubulin
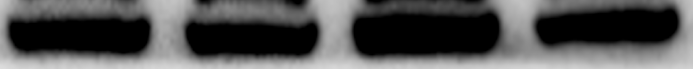


C S E N C S E N

P-IKB
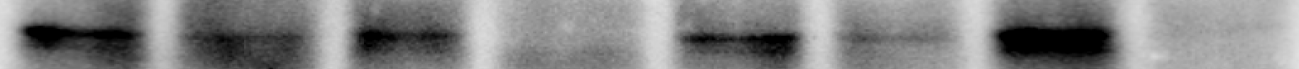


a-tubulin
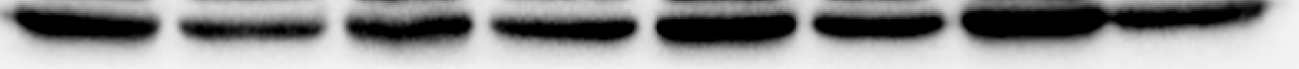


C S E N

P-IKB
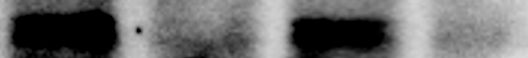


α-tubulin
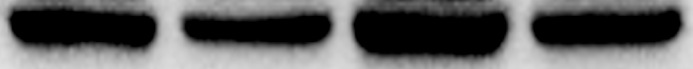


CON CD SD SR EA

GR
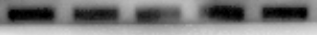


α-tubulin
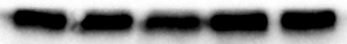


CON CD SD SR EA

GR
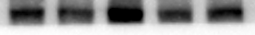


α-tubulin
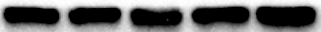


CON CD SD SR EA

GR
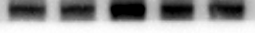


α-tubulin
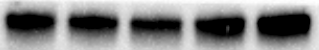


CON CD SD SR EA

GR
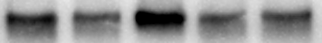


α-tubulin
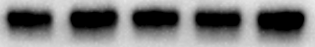


CON CD SD SR EA

GR
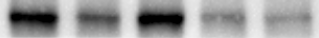


α-tubulin
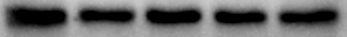


CON CD SD SR EA

GR
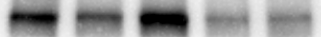


α-tubulin
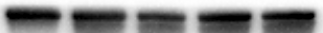


CON CD SD SR EA

P65
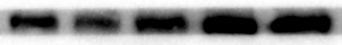


α-tubulin
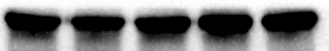


CON CD SD SR EA

P65
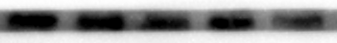


α-tubulin
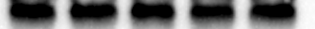


CON CD SD SR EA

P65
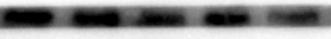


α-tubulin
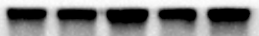


CON CD SD SR EA

P65
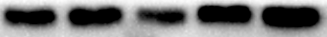


α-tubulin
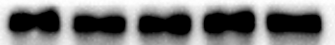


CON CD SD SR EA

P65
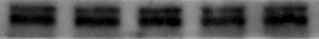


α-tubulin
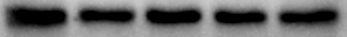


CON CD SD SR EA

P65
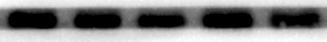


α-tubulin
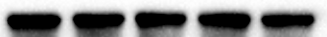


CON CD SD SR EA

P-P65
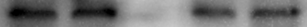


α-tubulin
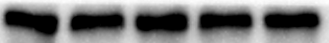


CON CD SD SR EA

P-P65
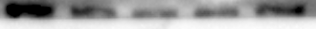


α-tubulin
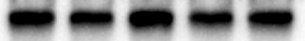


CON CD SD SR EA

P-P65
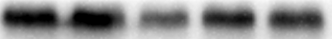


α-tubulin
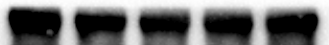


CON CD SD SR EA

P-P65
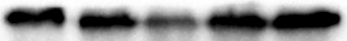


α-tubulin
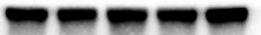


CON CD SD SR EA

P-P65
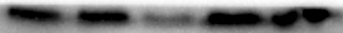


α-tubulin
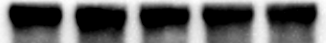


CON CD SD SR EA

P-P65
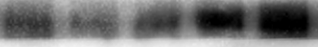


α-tubulin
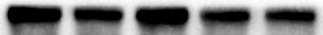

Supplement: Supplementary file 1 — Additional file 1. [file 12950_2023_354_MOESM1_ESM.docx]
